# Spatially-resolved Brillouin spectroscopy reveals biomechanical abnormalities in mild to advanced keratoconus in vivo

**DOI:** 10.1038/s41598-019-43811-5

**Published:** 2019-05-16

**Authors:** Peng Shao, Amira M. Eltony, Theo G. Seiler, Behrouz Tavakol, Roberto Pineda, Tobias Koller, Theo Seiler, Seok-Hyun Yun

**Affiliations:** 1 Harvard Medical School and Wellman Center for Photomedicine, Massachusetts General Hospital, Boston, MA 02114 USA; 2 Massachusetts Eye and Ear Infirmary, Boston, MA 02114 USA; 3 Institute for Refractive and Ophthalmic Surgery (IROC), Zürich, 8002 Switzerland; 4 Harvard-MIT Health Sciences and Technology, Cambridge, MA 02139 USA; 5 Universitätsklinik für Augenheilkunde, Inselspital, Bern, 3010 Switzerland

**Keywords:** Eye diseases, Biophotonics

## Abstract

Mounting evidence connects the biomechanical properties of tissues to the development of eye diseases such as keratoconus, a disease in which the cornea thins and bulges into a conical shape. However, measuring biomechanical changes *in vivo* with sufficient sensitivity for disease detection has proven challenging. Here, we demonstrate the diagnostic potential of Brillouin light-scattering microscopy, a modality that measures longitudinal mechanical modulus in tissues with high measurement sensitivity and spatial resolution. We have performed a study of 85 human subjects (93 eyes), consisting of 47 healthy volunteers and 38 keratoconus patients at differing stages of disease, ranging from stage I to stage IV. The Brillouin data *in vivo* reveal increasing biomechanical inhomogeneity in the cornea with keratoconus progression and biomechanical asymmetry between the left and right eyes at the onset of keratoconus. The receiver operating characteristic analysis of the stage-I patient data indicates that mean Brillouin shift of the cone performs better than corneal thickness and maximum curvature respectively. In conjunction with morphological patterns, Brillouin microscopy may add value for diagnosis of keratoconus and potentially for screening subjects at risk of complications prior to laser eye surgeries.

## Introduction

Growing evidence indicates that the biomechanical properties of ocular tissues can be diagnostic targets due to their association with various eye diseases and refractive errors^1^. The cornea is a prototypical example, requiring adequate mechanical stiffness to maintain the structure necessary for good vision in the presence of external mechanical stresses such as in-plane tension and intraocular pressure (IOP)^2^. The mechanical properties of the cornea stem from the intricate lattice of macromolecules, including collagen fibers and proteoglycans, making up the corneal stroma^3,4^. Disintegration of this structure alters the biomechanical properties of the stroma and shifts the overall mechanical homeostasis, potentially leading to vision-impairing morphological changes^5,6^.

Keratoconus (KC) is a bilateral, noninflammatory disease in which the normal prolate cornea locally thins and bulges into a conical shape^7^. Its etiology is not fully understood, but numerous experimental studies have suggested the importance of biomechanics. Disrupted collagen orientation^8–10^ and reduced mechanical moduli^2^ have been observed in KC explants and corneas that developed ectasia after laser-assisted *in situ* keratomileusis (LASIK). In addition, genetic and molecular studies have linked KC to disintegration of the collagen extracellular matrix^11–13^.

Current KC diagnosis is primarily focused on detecting abnormalities in corneal thickness and curvature. Advances in pachymetry and topography have greatly improved the diagnosis and treatment monitoring of KC. However, these morphological changes are thought to be secondary to biomechanical degeneration. Early-stage or ‘subclinical’ KC prior to the appearance of definitive morphological abnormality is considered a major risk factor for post refractive-surgery ectasia. The unmet clinical need for earlier KC detection and screening has motivated the development of increasingly sophisticated morphology-based metrics, specific genetic and molecular markers, and other diagnostic approaches such as measuring corneal biomechanics^1,14^.

Recently, two commercial instruments: the ocular response analyzer (ORA, Reichert Technologies Inc., USA) and the corneal visualization Scheimpflug technology (Corvis ST, OCULUS Optikgeräte GmbH, Germany), became available, which use a single air-puff to induce millimeter-scale deformation of the cornea and extract various viscoelastic parameters^15^. Numerous clinical studies have demonstrated the potential of these instruments for detecting moderate to severe KC^16–23^. Vinciguerra *et al*. have developed a biomechanical index based on the corneal thickness profile and several Corvis deformation parameters, which has shown potential for diagnosis of early-stage KC^24,25^. Recent ORA and Corvis studies have made progress towards screening for subclinical KC^26–28^. A drawback of the air-puff technique is that the corneal deformation is inherently dependent on additional factors such as IOP and the anatomical geometry of the eye^29^. Additionally, this technique can only measure the overall corneal stiffness, so is less sensitive to changes in a specific region of the cornea. Other promising techniques are under development, such as optical coherence elastography^30–33^ using various external stimuli^34–39^. However, the safety and feasibility of these techniques *in vivo* has yet to be tested.

We have previously demonstrated Brillouin light-scattering microscopy, which makes it possible to measure longitudinal mechanical modulus in tissues^40^. Unlike air-puff-based methods, the technology does not involve corneal deformation and can interrogate a specific region and depth in corneal tissue with optical resolution^41^. We have established the safety of this technology in humans and reported Brillouin elasticity maps of advanced KC patients’ corneas, providing *in vivo* evidence of spatial heterogeneity in their biomechanical properties^42^.

In this paper, we describe the largest clinical Brillouin studies to date, focusing on the early stages of KC. To detect subtle biomechanical changes in mild and moderate KC, we improved the measurement sensitivity and repeatability of Brillouin systems. A careful analysis of Brillouin frequency shifts measured *in vivo* led to several findings about previously unknown, biomechanical features in KC corneas and normal corneas. We present results that point to promising diagnostic metrics based on spatially-resolved biomechanical measurements.

## Results

### Development of *in vivo* Brillouin microscopes

**Figure 1:**
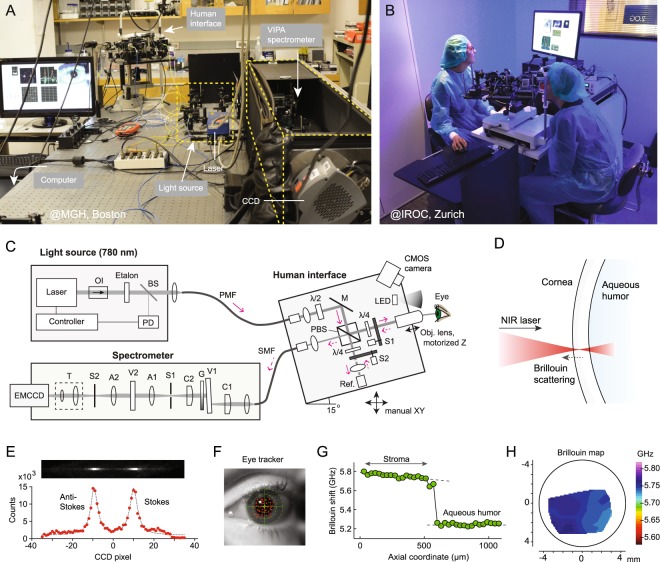
The Brillouin ocular analyzer system. (**A**) The tabletop Brillouin imaging system in the laboratory at Massachusetts General Hospital in Boston. (**B**) Brillouin measurement of a volunteer by an operator, using the portable Brillouin ocular scanner in the clinical setting at IROC in Zürich, Switzerland. (**C**) Schematic of the Brillouin imaging system, composed of three parts: a light source, a human scanning interface built on a modified slit-lamp platform, and a high-resolution, two-stage VIPA spectrometer consisting of two crossed-axis VIPA etalons. OI: optical isolator; PD: photodiode; BS: beam sampler; PMF: polarization maintaining single-mode fiber; SMF: single mode fiber; *λ*/2: half waveplate; *λ*/4: quarter-waveplate; M: mirror; Obj. L: objective lens; PBS: polarizing beam-splitter; S1/S2: optical shutters; Ref: reference materials; C1/C2: cylindrical lens; A1/A2: achromatic lens; S1/S2: shutters; V1/V2: VIPAs. (**D**) Probe beam geometry. (**E**) Representative Brillouin signals from the cornea recorded *in vivo* using the EMCCD camera in the spectrometer. (**F**) An eye-tracking camera view showing locations of axial scans (red dots). (**G**) A representative axial (depth) scan profile of measured frequency shifts across the cornea and anterior chamber of a healthy volunteer. (**H**) A typical Brillouin elasticity map of a normal cornea.

We have built two Brillouin microscopes with nearly identical optical designs. One tabletop system was used for studies at MGH in Boston (Fig. 1A). The other rack-mounted system was built at MGH and shipped to Zürich for studies at IROC (Fig. 1B). Compared to our previous instruments^42^, the new systems have several hardware and software upgrades to improve measurement sensitivity, stability and eye tracking, as well as mechanical robustness. Each system consists of a light source, human interface, spectrometer, and computer (Fig. 1C). The light source is a single-frequency tunable laser with its output spectrum locked to a near-infrared wavelength of ~780 nm and filtered using an etalon^43^. The laser light is coupled via a polarization-maintaining fiber to the human interface, in which polarization optics route the laser beam to the eye and direct backscattered light to a single-mode fiber. The spectrometer employs two-stage VIPA etalons and apodization filters to achieve a free-spectral range (FSR) of 16 GHz, a resolution of ~120 MHz, and an extinction efficiency of -65 dB. The optical power on the cornea is 3–5 mW (Fig. 1D), which is several times lower than the maximum permissible exposure level according to American National Standard Institutes (ANSI) guidelines (*Supplementary Information*). With this optical power applied to corneal tissues, an electron multiplying charge-coupled device (EMCCD) camera recorded a total of ~6,000 Brillouin photons per second. The Brillouin frequency shift, which is half the frequency difference between the Stokes and Anti-Stokes spectral peaks, is determined by curve fitting the recorded Brillouin spectra (Figs 1E and S2). With an EMCCD integration time of 0.2 s, we obtained a shot-noise-limited sensitivity of ~±8 MHz in Brillouin frequency shift measurement (Fig. S4). The spatial resolution was measured to be ~5 and ~35 µm in the transverse and axial directions, respectively (Fig. S5). The system employs temperature sensors and temperature-calibrated reference materials to attain short- and long-term repeatability of ~±10 MHz (Fig. S7). For corneal scans, an operator adjusts the human interface using a manual joystick to locate the focus at a desired location in front of the cornea while monitoring an eye-tracking video camera based on pupil detection (Fig. 1F). At each location, the operator starts a motorized axial scan to obtain a depth profile and calculate the mean Brillouin shift over the stroma (Fig. 1G). A typical 2D Brillouin elasticity map constructed from the mean Brillouin shifts measured at various scan locations using standard spatial interpolation is shown in Fig. 1H. Please refer to *Materials and Methods* and *Supplementary Information* for more technical details of the systems and data acquisition.

### Brillouin analysis of normal corneas *in vivo*

**Figure 2:**
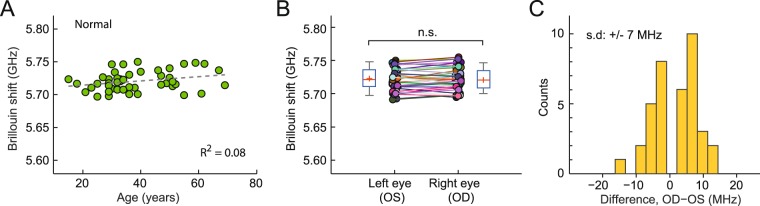
*In vivo* Brillouin measurements of normal corneas. (**A**) Brillouin frequency shifts measured at corneal centers in healthy subjects of various ages (n = 47, 39 ± 13 y/o, 51% female). Broken line depicts a linear regression fit with a slope of 0.3 × age MHz/year, with Pearson’s *p* = 0.06, *r*^2^ = 0.08. (**B**) Comparison of Brillouin shifts between the left (OS) and right (OD) eyes of each subject (n = 37). No statistically significant difference is found. (**C**) Distribution of OD-OS difference (mean: 0.0018 ± 0.007 GHz), from 37 subjects whose eyes were both scanned (38 ± 14 y/o, 60% female).

To establish a baseline for the normal population, we scanned healthy subjects with corneal thickness in a normal range of 495 to 600 µm, no irregular astigmatism, and no history of ophthalmic pathology or surgery. For each subject, a mean central Brillouin shift was obtained from 5 axial scans in a central region within a 2-mm radius from the pupil center. The measured values from 47 healthy subjects (age: 39 ± 13 y/o, 51% female, one eye per subject with left/right chosen at random) ranged from 5.69 to 5.76 GHz with a standard deviation (SD) of 15 MHz. When analyzed as a function of age (Fig. 2A), the Brillouin shift values tend to increase with age at a nominal slope of ~3 MHz per decade, but the correlation was not statistically significant. 37 of the 47 subjects had both of their eyes scanned. The mean Brillouin shifts were nearly identical within the instrument’s resolution for the left and right eyes: 5.721 ± 0.017 versus 5.723 ± 0.017 GHz (Fig. 2B). The difference between the left and right eyes of each individual subject makes a narrow, Gaussian-like distribution centered at 1.8 MHz (mean) (Fig. 2C). The bilateral symmetry between the two eyes of healthy subjects is remarkable and in contrast with the large interpersonal variability of ± 15 MHz.

### Brillouin measurements of keratoconus patients at different stages of disease

**Figure 3:**
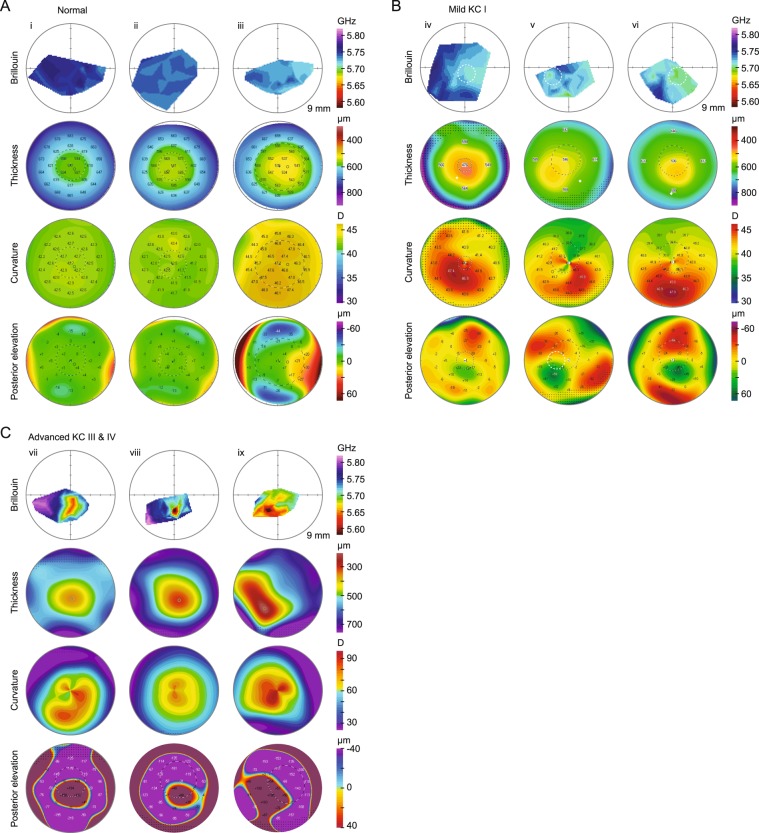
Representative Brillouin images of corneas *in vivo*. (**A**) normal corneas (subjects i to iii), (**B**) corneas diagnosed with keratoconus in Stage I (iv-vi), and (**C**) corneas with severe keratoconus in Stage III or IV (vii-ix). The circles (black) indicate an area of 9 mm in diameter centered at the pupil center. Rows 2–4 are topographical images of the corneas, showing corneal thickness, sagittal curvature, and posterior surface elevation. (Note the difference in color maps and ranges due to difference in clinical Scheimpflug imaging systems).

To investigate KC, subjects were scanned who have been diagnosed with KC by corneal specialists. The patients were grouped into 4 stages of severity according to the Amsler-Krumeich classification^44^ using quantitative criteria, namely refractive error, best spectacle-corrected visual acuity, minimum corneal thickness, and maximum curvature (K-max). For each cornea, pachymetry and topography images identified a “cone” region, which we define as the area within a 1 mm radius from the thinnest point. Brillouin scans were conducted at 20 to 40 lateral locations to generate a Brillouin map of the cornea. A few representative Brillouin maps are shown in Fig. 3, comparison to Brillouin maps of normal corneas (Fig. 3A). For advanced KC corneas in stage III or IV, Brillouin maps showed considerable non-uniformity compared to normal corneas (Fig. 3C). The cones, defined herein by the thinnest points, are approximately coincident with the bulging areas of abnormal curvature and surface elevation, and these areas are characterized by low Brillouin frequency shift values, distinctly different by as much as 100–200 MHz compared to healthy corneas. Stage-I corneas are characterized by mild morphological anomalies, and the thinnest points do not always overlap with the locations of maximum curvature or posterior elevation. Compared to stage-III-IV cases, state-I corneas exhibit much more uniform Brillouin maps, and the regions of lower Brillouin values are less pronounced (Fig. 3B).

**Figure 4:**
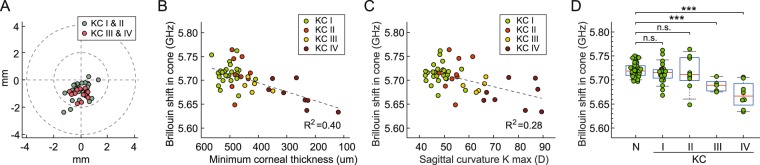
Correlation between Brillouin frequency values and KC severity. (**A**) Locations of the thinnest points identified by pachymetry in patients diagnosed with KC stage I or II (n = 34, 36 ± 11 y/o; 47% female) or KC stage III or IV (n = 12, 46 ± 14 y/o; 75% female). (**B**) Correlation of Brillouin shift with corneal thickness. (**C**) Correlation of Brillouin shift with sagittal curvature. Dashed lines: linear fits. (**D**) Comparison of Brillouin frequency values of normal subjects and KC patients at different stages: I (n = 25, 36 ± 11 y/o, 46% female), II (n = 9, 45 ± 13 y/o, 44% female), III (n = 4, 47 ± 12 y/o, 40% female) and IV (n = 8, 45 ± 15 y/o, 25% female). n.s.: non-significant, ***p < 0.001, by unpaired *t*-test.

The locations of minimum thickness, i.e. cone centers, are distributed predominantly in the center-temporal region (Fig. 4A). The XY coordinate in mm of the center of the distribution was (0.45, -0.85) for stages I and II (n = 34, 36 ± 11 y/o, 47% female) and (0.48, -1.2) for stages III and IV (n = 12, 46 ± 14 y/o, 75% female). The data suggest that the cone location within an individual may not change much as KC progresses. From the Brillouin maps, we calculated the mean Brillouin frequency shifts in the cone regions and plotted the data as a function of minimum corneal thickness (Fig. 4B) and maximum anterior sagittal curvature, K max (Fig. 4C). Weak to moderate trends of decreasing Brillouin frequency shifts with increasing KC severity were measured, with coefficients of determination for linear regression being R^2^ = 0.40 for corneal thickness and R^2^ = 0.28 for K-max. The graph in Fig. 4D shows the distributions of Brillouin frequency shift values in cone regions of KC patients in comparison to Brillouin values measured in central regions of normal patients (within a 1-mm radius from the pupil center). While the reduction of Brillouin values is evident for advanced KC, no statistically significant difference was observed between stage-I-II KC and normal corneas. The mean Brillouin values of the stage-I and -II groups are lower than the healthy group by 3 and 7 MHz, respectively. Nonetheless, the observed null correlation results from large interpersonal variability within each group. Notably, the subject-to-subject variation in stage I and II patients is greater than the intrinsic interpersonal difference among healthy subjects.

### Analysis of regional biomechanical variation in keratoconus corneas

**Figure 5:**
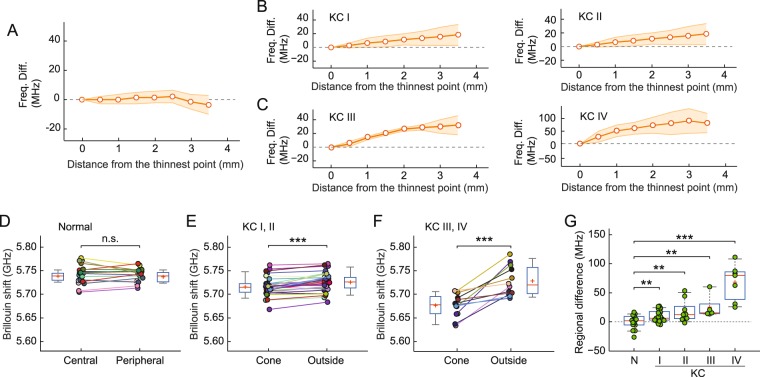
Spatial variation of Brillouin frequency value across corneas. (**A**–**C**) Lateral variation of Brillouin shifts in: (**A**) normal subjects, (**B**) stage-I KC patients, (**C**) stage-II KC patients. Circles and shaded regions represent average and SD of 4 to 16 maps. (**D**–**F**) Pairwise comparison of Brillouin values in corneal centers or cones to Brillouin values in peripheral regions, for (**D**) normal subjects (n = 16, 39 ± 10 y/o, 25% female): 5.738 ± 0.010 GHz (center) vs. 5.737 ± 0.011 GHz (peripheral), (**E**) Stage I-II KC: 5.716 ± 0.020 GHz (cone) vs. 5.726 ± 0.020 GHz (outside cone), (**F**) Stage III-IV KC patients: 5.677 ± 0.024 (cone) vs. 5.728 ± 0.032 GHz (outside cone). **p < 0.01, ***p < 0.001, by paired *t*-test. (**G**) Difference in Brillouin shift measured in the outside-cone (peripheral) versus cone (central) regions for KC and normal (N) groups. n.s.: non-significant, **p < 0.01, ***p < 0.001, by unpaired *t*-test.

The observation of large interpersonal variability in both normal and KC corneas prompted us to seek metrics that are less subject to individual variations. We hypothesized that most of the variability stems from natural personal differences in the composition, collagen organization, and/or hydration level of corneal tissues. We further reasoned that these factors would likely affect Brillouin values uniformly across the entire cornea and therefore can be canceled out in differential metrics, such as regional difference within the cornea. From Brillouin maps, we found that, whereas normal corneas exhibit relatively uniform Brillouin values across their about-4-mm-wide scanned areas (Fig. 5A), the Brillouin shifts in KC corneas have a clear tendency to increase linearly with the distance from the cone in all stages, including stages I and II (Fig. 5B,C). The slope of regional variation increased with the severity of KC (Fig. S10). We compared the mean Brillouin frequency shift in the cone region versus outside-cone region—defined by all scanned points outside a 3-mm radius from the thinnest point—for each cornea. For healthy subjects, central and peripheral Brillouin values are not different (Fig. 5D, n = 16, 39 ± 10 y/o, 25% female). By contrast, highly significant differences (p < 0.001) were found between cone and outside-cone regions for all stages of KC (Fig. 5E,F). Figure 5G shows regional variations or the differences of outside- and inside-cone values for all groups. All KC groups exhibit higher regional differences than the normal group. However, the statistical significance is moderate (p < 0.01) except for stage IV, with considerable overlaps of Brillouin values with the normal group.

### Analysis of bilateral symmetry in KC corneas

**Figure 6:**
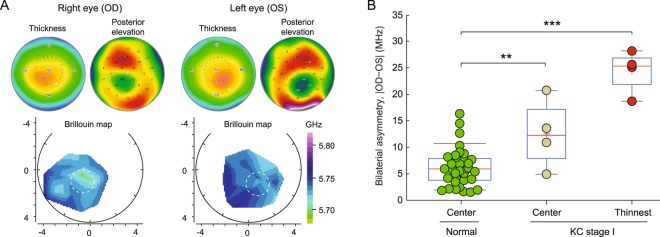
Bilateral asymmetry in early-stage KC. (**A**) Brillouin, corneal thickness and posterior elevation maps of the right and left eyes of a stage-I KC patient. Broken red circle indicates a 1-mm-radius cone region centered at the thinnest point. (**B**) Absolute difference in Brillouin frequency values between left and right eyes, measured at the corneal center for normal subjects (0.006 ± 0.004 GHz), the corneal center for stage-I KC patients (0.013 ± 0.007 GHz), and at the thinnest points for stage-I KC patients (0.024 ± 0.004 GHz). Normal subjects: n = 47, 39 ± 13 y/o, 51% female. Stage I KC patients: n = 4, 38 ± 5 y/o, 50% female. **p < 0.01, ***p < 0.001, by unpaired t-test.

KC is known to be bilateral, but often asymmetric. The high degree of left-to-right symmetry in central Brillouin values in healthy subjects motivated us to analyze bilateral symmetry for KC corneas. Representative Brillouin maps from a patient with stage-I KC in both corneas are shown in Fig. 6A. The thickness and posterior elevation maps share symmetric, but not identical, features. Asymmetry in the Brillouin maps is apparent although subtle. To quantify the asymmetry, we calculated the difference in Brillouin frequency shifts between left (OS) and right (OD) eyes in two corresponding locations: near the pupil center and in the cone. The asymmetry in Brillouin values in the central regions of KC corneas was moderately higher (p < 0.01, n = 4, 38 ± 5 y/o, 50% female) than in normal corneas (Fig. 6B). Remarkably, the Brillouin asymmetry between the left and right cone regions, which are identified using the thickness maps, is significantly larger than for the normal group (Fig. 6B). The mean bilateral difference in the stage-I cones was 24 MHz, well separated from the normal population that is predominantly below 12 MHz. Despite the relatively small sample size, the data are compelling and reveal that bilateral symmetry may be a promising metric for diagnosis of early-stage KC. The narrow distribution of the data suggests that the compounding factors that are responsible for the subject-to-subject variability in Brillouin values are equally present in both eyes and, therefore, cancelled out in this differential metric.

## Discussion

The magnitude of the optical frequency shift that we measure is proportional to the velocity of longitudinal acoustic waves in the tissue, which is in turn related to the square root of longitudinal modulus of elasticity^45^. For viscoelastic materials such as tissues, longitudinal modulus is nearly equal to bulk modulus, or the material’s resistance to volume change. Because most biological tissues are ~70% water by volume, they are nearly incompressible and exhibit high longitudinal modulus, in the range of 2.5 to 3.5 GPa^46^, producing Brillouin frequencies of 5.69 to 5.77 GHz in corneas^42^ and up to 6.1 GHz in crystalline lenses^47^ (at an optical wavelength of 780 nm). The bulk modulus of aqueous humor, which is nearly the same as that of water, is 2.2 GPa, for which we measured a Brillouin shift of 5.25 GHz (Fig. 1G). The higher longitudinal-modulus of corneal tissue compared to water derives from the contribution of the other constituent matter such as ions and proteins, that make the tissue less compressible. Brillouin spectroscopy has been shown to be sensitive to small differences in molecular composition, concentration (or water content)^46^, and photochemical crosslinking^48,49^. Brillouin measurement may be less sensitive to processes such as pure polymeric rearrangement or changes that are predominantly entropic, but relatively more sensitive to processes involving changes in molecular concentration and composition.

In contrast to bulk modulus, shear or Young’s modulus characterizes a material’s response to stress under free boundary conditions. Since soft matter is deformed relatively easily at constant volume, its shear modulus is orders-of-magnitude smaller than its bulk modulus. The shear modulus measured by mechanical methods at low deformation frequencies (<100 Hz) is typically ~250 kPa for normal corneal tissue. Although longitudinal and shear moduli are in principle two independent quantities and can have vastly different magnitudes, empirical data obtained with polymers, hydrogels, and various tissues including corneas have shown reasonably high, quantitative correlation between Brillouin frequency shift and quasi-static shear modulus^41,42,50^. This measurement result suggests that in practice for corneal tissues, natural or pathologic processes that cause shear modulus to increase (or decrease) are likely to cause Brillouin shifts to increase (or decrease).

The Brillouin data from normal adult corneas *in vivo* showed a modest age-dependence with a slope of ~3 MHz/decade. Unexpectedly, we observed relatively large subject-to-subject differences (±15 MHz SD) even among age-matched subjects (Fig. 2A). Given the interpersonal variability, the rather small age dependence is surprising, as corneal surgeons often experience less soft and pliable corneas in old patients compared to young. Numerous *ex vivo* mechanical measurements have reported increasing trends of Shear and Young’s moduli with age^51–53^. About 12% increase of Young’s modulus per decade has been estimated^53^. Employing an often-used empirical log-log conversion factor of 60:1, the slope of 12% per decade in Young’s modulus corresponds to, approximately, 0.2% per decade in longitudinal modulus, or 12 MHz/decade in Brillouin frequency (*Supplementary Information*). Previously, electron microscopy has revealed thickening of collagen fibrils^54^ and decrease of their inter-fibrillar spacing^55^ with age. Such changes in microstructure^56^, as well as age-related increase of glycation-mediated collagen crosslinking^57^, are thought to elevate corneal stiffness over age^56^. The apparent discrepancy between our measurement and previous *ex vivo* measurements (as well as surgeons’ perception by *in vivo* palpation) has yet to be resolved. It is interesting to note that *in vivo* measurements using ORA found no increase or even small decline of corneal hysteresis and corneal resistance factor with age^21,58–60^.

We suspect that the interpersonal variability stems from natural differences in the physiological conditions of the cornea. For example, Brillouin shift is sensitive to the water content in hydrogels and, therefore, hydration level of corneal tissues^61^. The hydration level is known to differ individually and fluctuate throughout the day^62^. This hydration variation may contribute to the measured interpersonal variability. The Brillouin shift of tissue is also sensitive to body temperature, as expected from a temperature dependence of 7.4 MHz/°C for water (Fig. S6). It is worth noting that ocular surface temperature was found to be negatively correlated with age^63^, which may contribute to the little-to-no age dependence we observed in our study. In future, it would be preferable to record body temperature and apply a correction factor to the measured Brillouin values. Since these physiological factors affect globally the entire cornea, we had expected smaller variability for differential metrics. Indeed, we measured substantially smaller “intra-personal” differences between left and right eyes (Fig. 2B) and between the central and peripheral regions of each cornea (Fig. 5D).

Variability in corneal biomechanics across individuals can introduce significant errors in applanation-tonometry IOP readings^64^. Hence, individual differences in Brillouin value may be used to apply a “biomechanics correction” to improve the accuracy of IOP measurements. Individual differences in Brillouin value may also provide useful input for refractive treatments: it is known that corneal hydration affects the excimer laser ablation rate in LASIK surgeries^65,66^, and personal biomechanical differences are thought to explain the variability in refractive outcomes following cataract and astigmatic correction keratotomy (AK) surgeries^67^. It may be possible to individually tailor ablation parameters or AK nomograms based on Brillouin measurements made prior to treatment.

In the present study of KC patients, we confirmed our previous finding that the cones in advanced KC corneas are clearly visible in Brillouin maps as regions of significantly reduced Brillouin shift (Fig. 4B)^42^. However, the correspondence between Brillouin maps and pachymetry and topography is less clear in early-stage KC corneas, and Brillouin shifts correlate weakly with morphological parameters such as thickness and curvature (Fig. 4B,C). We found no statistical difference between stage-I-II KC patients and healthy subjects (Fig. 4D). The Brillouin values within stage-I and II groups each exhibited larger inter-subject variation than the normal group. The large variations might originate from the physiological factors discussed above and, possibly, additional factors associated with KC pathogenesis.

The diagnosis of KC in this study was performed by corneal experts at IROC using proprietary criteria based on corneal pachymetry and topography maps. We calculated receiver operating characteristic (ROC) curves for two morphological parameters—minimum corneal thickness and maximum sagittal curvature—and two Brillouin parameters—mean Brillouin shift of the cone and regional Brillouin difference—using the data from stage-I patients (n = 25) data (Fig. S11). The area under the curve (AUC) values of the Brillouin parameters were 0.92 for mean Brillouin and 0.85 for regional difference, which are higher or comparable to 0.87 for maximum curvature and 0.77 for minimum thickness. This result supports that the Brillouin parameters may add value for diagnosis of early-stage KC. Future studies with larger patient numbers and more accurate, comprehensive corneal mapping is warranted to validate this finding.

The distinction between early-stage KC and normal groups was more pronounced in the analysis of regional differences between cone and outside-cone regions. In most stage-I and -II subjects and all stage-III and IV subjects, the cone regions had lower Brillouin shifts than the peripheral regions. This supports the long-held hypothesis that the degeneration of corneal tissues occurs focally at the cones^14^. Given moderate statistical significance (p < 0.01) differentiating stage-I from normal values, the regional-difference parameter has potential to be a useful metric to characterize early-stage KC. However, the two-point comparison alone is unlikely to be diagnostic of KC, considering the overlap of data between different groups (Fig. 5G). With more accurate, comprehensive mapping of the cornea in future, more sophisticated metrics based on specific spatial patterns in Brillouin maps may be developed. Nevertheless, the regional differences could still be of significant importance for clinical practice. One potential application could be personalized CXL treatment planning and outcome efficacy monitoring^68^.

Another promising metric we have found is the difference in Brillouin shifts between individual left and right eyes. The bilateral asymmetry measured in stage-I KC patients is clearly separated from that of normal corneas, and reflects the clinical experience that in many keratoconus patients one eye presents a more advanced stage of disease relative to the other^69^. More investigation of this metric in a larger-scale study is warranted, which may include patients with only one eye in stage-I KC and a subclinical or KC suspect contralateral eye. The bilateral asymmetry indices of healthy subjects form a normal distribution with width limited by the system sensitivity. With enhanced Brillouin signals through longer data acquisition time, the distribution in the healthy population may turn out to be narrower. The bilateral asymmetry index may prove useful for identifying patients at an earlier stage or those at risk of developing KC. If so, this index could be used to develop new diagnostic criteria in combination with, or even beyond the current morphology-based gold standard. It is worthwhile to note that it is the bilateral asymmetry measured at the thinnest point that showed the sharp distinction. The asymmetry index measured at the pupil center showed only moderate statistical difference from the normal population (Fig. 5B). This consistently supports the hypothesis of focal weakening in the development of KC^14^.

An interesting future project would be to compare Brillouin measurements to those obtained from air-puff-based instruments, given the recent success of these methods^25–28^. Though these methods are essentially measuring different physical properties of the tissue, correlation may be found between data acquired with these methods, such as corneal hysteresis or the Corvis ST biomechanical index, and certain Brillouin metrics, such as regional difference.

Besides corneas, other ocular tissues such as the crystalline lens and sclera are thought to present a high degree of symmetry in their healthy states. In fact, in our previous study of crystalline lenses *in vivo*, we have measured large interpersonal variability, as much as ± 60 MHz in age-matched groups, as opposed to small differences between left and right lenses within the measurement uncertainty of ± 10 MHz^47^. Brillouin-based bilateral asymmetry measurement has potential to be a general, useful technique for the detection of subtle pathological anomalies and drug-induced changes in the biomechanical properties of the lens and sclera.

The current Brillouin systems require a data acquisition time of >0.2 s per depth point and ~12 s for a single axial scan. A higher data acquisition speed is desirable for clinical use. The total number of Brillouin scattered photons that were collected and analyzed at the spectrometer was about 3 × 10^−13^ of the number of laser photons incident on the corneal surface. The theoretical maximum collection efficiency of Brillouin light scattering in an on-axis confocal configuration is estimated to be $\frac{2{\pi }^{2}}{{\lambda }^{3}}\frac{{({n}_{s}^{2}-1)}^{2}}{K}{k}_{B}T$, where *k*_*B*_*T* = 4.23 × 10^−21^ J at the cornea temperature, *n*_*s*_ is refractive index, *K* is bulk modulus, and *λ* is optical wavelength (*Supplementary Information*). For corneal tissues, the efficiency is ~5 × 10^−11^. There is room to increase the Brillouin signal by as much as 10-fold by reducing optical loss in the detection path.

In conclusion, these clinical results have demonstrated the potential of Brillouin light scattering spectroscopy for corneal examinations, particularly for detection and treatment monitoring of KC patients. The most promising metric for KC-discrimination are based on focal weakening, a finding made possible by harnessing the intrinsic ability of Brillouin technology to measure local biomechanical properties of tissues with high spatial resolution and sensitivity. Our results suggest that spatially-resolving Brillouin spectroscopy may also prove useful in other applications, including screening subjects at risk of ectasia prior to refractive keratectomy surgeries^70–74^, optimizing corneal incision parameters in cataract and astigmatic correction keratotomy surgeries by taking individual biomechanics into account^67^, and treatment-parameter optimization and outcome evaluation for customized CXL treatment^68^. Brillouin measurements may also be used to improve the accuracy of basic examinations such as intraocular pressure measurement^64^.

## Methods

### Study design

The objective of this study was to assess the feasibility of using Brillouin spectroscopy for clinical characterization of human corneal biomechanics. Specifically, to evaluate the potential of Brillouin spectroscopy for use in detection and treatment of keratoconus. For this purpose, we examined the interpersonal variability, age-dependence, regional heterogeneity, and bilateral asymmetry in Brillouin values among normal corneas, keratoconus corneas of different severity, and corneas with history of collagen crosslinking treatment.

The general inclusion criteria permitted subjects with clear-enough cornea and media to permit imaging, and who fulfilled the specific study group inclusion/exclusion criteria. Excluded from this study were monocular volunteers. The normal cornea group (n = 47, 39 ± 13 y/o, 51% female) included healthy subjects with normal appearing corneas consistent with the general inclusion/exclusion criteria, with less than ± 3 diopters refractive error, normal corneal thickness (495 to 600 µm), normal corneal topography (no asymmetric or irregular astigmatism, no skewed axis), no corneal pathology, and no history of eye diseases, except presbyopia and/or cataract. The keratoconus group (n = 46, 39 ± 13 y/o, 43% female) included subjects with irregular corneas determined by distorted keratometry mires or irregularities in Scheimpflug photography and slit-lamp biomicroscopic signs such as Vogt’s striae or Fleischer’s ring or corneal scarring consistent with keratoconus, aged from 15–60 y/o. This group included patients diagnosed with KC stage I (n = 25, 36 ± 11 y/o, 46% female), II (n = 9, 45 ± 13 y/o, 44% female), III (n = 4, 47 ± 12 y/o, 40% female) and IV (n = 8, 45 ± 15 y/o, 25% female).

Sample sizes for each group were chosen in advance based on a power analysis. We used preliminary data to estimate the sample size required to establish a correlation between corneal stiffness and progression of the disease and subject age, with >90% power.

Studies were performed at two locations: at the Institute for Refractive and Ophthalmic Surgery (IROC) in Zürich, Switzerland, and at Massachusetts Eye and Ear Infirmary (MEEI) and Massachusetts General Hospital (MGH) in Boston, USA, following approval from the Institutional Review Board (IRB) of Partners HealthCare, the Partners Human Research Committee (PHRC) and the Institutional Review Board of IROC, Zürich. Informed consent was obtained from every patient before imaging. All methods were performed in accordance with the relevant guidelines and regulations. The clinical trial “A Study to Test the Potential of Brillouin Microscopy for Biomechanical Properties Measurements in Human Cornea” is registered at www.clinicaltrials.gov (National Clinical Trial Identifier: NCT03220529).

### Brillouin data acquisition

During Brillouin measurement, the subject sits with their chin and forehead resting in the human interface headrest, directing their gaze towards a fixation target. For healthy subjects with normal corneas, 5 axial scans are taken within a ∅2 mm zone in the central cornea. To create a Brillouin map of the cornea, 20–40 axial scans are taken at different locations laterally across the cornea, usually within the central ∅6 mm zone. Light power was calibrated to be 5 mW on the corneal surface. Each axial scan comprises 40 points separated by a step size of 30 μm, spanning air to cornea to aqueous humor. The EMCCD exposure time for each step is typically 300 ms, resulting in a total axial scan time of about 12 s. Following each axial scan, temperature-corrected calibration spectra are taken using known reference materials (polystyrene and water) to determine the dispersion rate (in GHz/pixel) of the spectrum pattern acquired from the cornea, and thus the corresponding Brillouin frequency shift (Fig. S3). From the calibrated axial scan data, points acquired within the corneal stroma are extracted and averaged to yield a single value for each lateral scan location (Fig. 1G). Linear interpolation between these lateral measurement points is then used to create the final 2D Brillouin map (Fig. 1H). Data processing was conducted using MATLAB (Mathworks, Inc.).

### Corneal topographies

The corneal topographies were generated using a commercial Scheimpflug camera (Pentacam HR 70700, OCULUS, Wetzlar, Germany) with thickness mapping and posterior elevation characterization. Patients were asked not to use contact lenses in the two weeks prior to each examination.

### Data analysis

To process a single axial scan, we first determine the rate of spectral dispersion using the included calibration spectrum (see Fig. S3). With this information, we can then determine the Brillouin frequency shift at each of the (typically) 40 scan points spanning air to cornea to aqueous humor. We then segment this Brillouin shift versus axial depth data, based on Brillouin value and other characteristic signal features, in order to identify and extract only points within the corneal stroma. Finally, we average the Brillouin shift measured across all stroma points to obtain a single Brillouin value for the axial scan.

To create a Brillouin map of the cornea, we use standard linear interpolation to connect Brillouin values from 20–40 axial scans at different lateral locations. To analyze the spatial variation in Brillouin values across the cornea, we define various regions of interest (see Fig. S9 for a graphical illustration of these zones). The central region is defined as the zone within a 1-mm radius around the pupil center; the peripheral region is the area with a distance >3 mm from the pupil center. In keratoconic corneas, the ‘cone region’ is defined as the area within a 1 mm radius of the thinnest point (the ‘cone center’) identified using corneal topography, and the ‘outside-cone region’ refers to the area >3 mm from the cone center.

### Statistical analysis

Custom MATLAB software was used for data processing. Statistical analysis was carried out using the Statistics toolbox in MATLAB. To examine the significance of differences observed in the mean Brillouin values of different groups of subjects (such as comparing normal versus KC stage I) we used unpaired, two-tailed *t*-tests. To evaluate the significance of differences observed within a single cornea (such as comparing one region to another) we used paired, two-tailed *t*-tests. Data were considered significant if p-values were less than 0.05 (95% confidence intervals).

## Data Availability

The data that support the findings of this study are available from the corresponding author upon reasonable request.

## Supplementary information

Spatially-resolved Brillouin spectroscopy reveals biomechanical abnormalities in mild to advanced keratoconus in vivo
