# Supplementary material for: Spatially-resolved Brillouin spectroscopy reveals biomechanical abnormalities in mild to advanced keratoconus in vivo
Source: Sci Rep. 2019 May 16;9:7467. doi: 10.1038/s41598-019-43811-5 (PMC6522517; doi:10.1038/s41598-019-43811-5)
Supplement: Supplementary file 1 — Spatially-resolved Brillouin spectroscopy reveals biomechanical abnormalities in mild to advanced keratoconus in vivo [file 41598_2019_43811_MOESM1_ESM.pdf]

Supplementary Information

Spatially-resolved Brillouin spectroscopy reveals biomechanical abnormalities in mild to advanced keratoconus in vivo

Peng Shao[1†], Amira M. Eltony[1†], Theo G. Seiler[1,3,4], Behrouz Tavakol[1], Roberto Pineda[2], Tobias Koller[3], Theo Seiler[3*], Seok Hyun Yun[1,5**]

[1] Harvard Medical School and Wellman Center for Photomedicine, Massachusetts General Hospital, Boston, MA 02114, USA

[2] Massachusetts Eye and Ear Infirmary, Boston, MA, USA

[3] Institute for Refractive and Ophthalmic Surgery (IROC), Zurich, Switzerland

[4] Universitätsklinik für Augenheilkunde, Inselspital, Bern 3010, Switzerland

[5] Harvard-MIT Health Sciences and Technology, Cambridge, MA, USA

† equal contribution

* theo@seiler.tv, ** syun@hms.harvard.edu

Relation of Brillouin frequency shift and longitudinal elastic modulus

Spontaneous Brillouin scattering is the scattering of light from acoustic phonons (i.e. propagating density waves originating from intrinsic thermal fluctuations) [1,2]. Light scatters from acoustic waves that are phase-matched, resulting in a negative (Stokes) and positive (Anti-Stokes) Doppler frequency shift of the light by the frequency of the mechanical waves. It is the (absolute) magnitude of Brillouin frequency shift that we measure with our optical spectrometer. Although both shear and longitudinal waves are present in the tissue, our Brillouin instrument is configured to measure 180º back-scattering from longitudinal waves only (Fig. 1). In this back-scattering configuration, the Brillouin frequency shift Ω is related to the acoustic speed V, via $V = \Omega \frac{\lambda}{2n}$, where λ is the wavelength of the light, and n is the refractive index of the tissue. The acoustic speed is itself a function of the longitudinal elastic modulus M through the relation $V = \sqrt{M/\rho}$, where ρ is the mass density of the tissue.

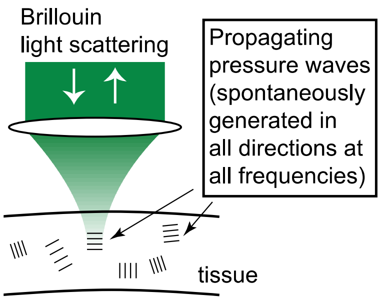

Fig. S1: Brillouin light scattering in the cornea.

From this equation, we get $\frac{\Delta\Omega}{\Omega} = \frac{\Delta M}{2M} + \frac{\Delta n}{n} - \frac{\Delta\rho}{2\rho}$. Based on published data [3,4], we estimate $\frac{\rho}{n^2} \approx$ 0.565 to 0.5635 g/mL, varying <0.3% within normal corneas. Therefore, the last two terms are nearly canceled out. In general, the acoustic speed has directional dependence and M is a tensor. If we approximate the corneal tissues as a mechanically isotropic material, the relationship between different mechanical moduli is as follows: $G = K\frac{3(1-2\nu)}{(1+\nu)}$; $M = K + \frac{4G}{3}$, where G is shear modulus, K is bulk modulus, and ν is Poisson's ratio. K is an inverse of volume compressibility. In tissues, M is approximately equal to K. The typical value of longitudinal modulus is several GPa whereas that of shear modulus of tissues is in a 10-100 kPa range [5].

Empirically, we have previously observed a relatively high correlation between longitudinal modulus (M) and quasi-static shear modulus (G) in *ex vivo* porcine corneas (Fig. S2) when fit to a log-log linear curve: $\log(M) = c \log(G) + d$, with fitting parameters c = 0.033 and d = 9.31 (Scarcelli G and Yun

SH, in Biomechanics of the Eye, 2018 Kugler Publications, pp. 159-168). For *ex vivo* samples, the effect of swelling may contribute to the apparent correlation between longitudinal and shear moduli. That is, tissues with low shear modulus tend to swell more, and the increased swelling reduces longitudinal modulus. We do not yet know to what extent the observed empirical relation would hold in the physiological condition *in vivo*, in which tissue hydration is regulated. Regardless, the Brillouin frequency shift itself is a convenient metric directly measurable with Brillouin light scattering spectroscopy. In this paper, we report our measurements as Brillouin frequency shifts in GHz.

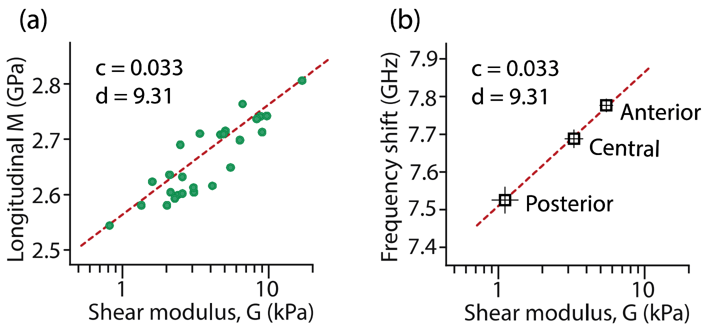

Fig. S2: Empirical quantitative relationship between Brillouin modulus (frequency shift) and shear modulus (G). (a) Comparison data for thin flaps of porcine corneal tissue *ex vivo* (each data point corresponds to one thin flap). Cornea tissue was cut with a biopsy punch to retrieve thin flaps from the anterior, central, and posterior regions. Shear modulus of the thin flaps was measured using a stress-controlled rheometer (ARG2, TA Instruments) at 0.5 Hz with 0.1% strain amplitude. High correlation (R = 0.9) was obtained with c = 0.033 and d = 9.31. (b) Data from (a) grouped by corneal region and plotted versus Brillouin frequency shift measured at a wavelength of 532 nm.

The magnitude of Brillouin scattering

The scattering cross section for Brillouin scattering in an isotropic liquid is given by [6]

$$\sigma = \frac{\pi^2 V}{2\lambda^4}\left(\frac{\gamma^2}{K}\right) k_B T(1 + \cos^2\theta)$$

where λ is the optical wavelength, γ is the adiabatic electrostriction constant, K is the adiabatic bulk modulus (at the Brillouin shift frequency), k_B is Boltzmann constant, T is the temperature, V is the volume of the interaction region, and θ is the scattering angle. The adiabatic electrostriction constant is a dimensionless measure of the change of the refractive index upon density variation, i.e. $\gamma = \rho(\frac{\partial\varepsilon}{\partial\rho})$, where

ε is dielectric constant and ρ density. To a good approximation, γ is equal to $\varepsilon - 1 = n^2 - 1$. The normalized magnitude of scattering is therefore given by

$$S = \frac{\sigma}{A} = \frac{\pi^2 L}{2\lambda^4}\left(\frac{\gamma^2}{K}\right) k_B T(1 + \cos^2\theta)$$

where A and L are the area and length of the interaction region, respectively. We consider a Gaussian probe beam focused to a spot with numerical aperture, NA, in free space. Then, $L \approx \frac{2\lambda}{\pi(NA)^2}$. In an on-axis, backward confocal geometry (θ close to 180°), only the scattered light within the solid angle given by the numerical aperture is collected. Integrating the angle-dependent term, $(1 + \cos^2\theta)$, over this range yields $\approx 2\pi(NA)^2$. Therefore, the total amount of light collected as a fraction of the laser photons incident on the cornea is:

$$S_{collected} = \frac{2\pi^2}{\lambda^3}\left(\frac{\gamma^2}{K}\right) k_B T$$

For cornea tissues, we use n = 1.376, $K = 2.6 \times 10^9$ J/m^3, $k_B T = 4.23 \times 10^{-21}$ J (cornea temperature ~33°C), and $\lambda = 0.78 \times 10^{-6}$ m, and obtain $S_{collected} = 5.4 \times 10^{-11}$. The scattering efficiency of measurement is independent of NA. It is interesting to find that the collection efficiency is proportional to λ^{-3}, while the total amount of scattering is proportional to λ^{-4} as is well known for Rayleigh and Brillouin scattering.

Brillouin microscopes

Light source

The light source is a single-frequency, external cavity diode laser (ECDL) with a center wavelength of 780 nm (Vantage TLB-7113, New Focus). Amplified spontaneous emission (ASE) present in the ECDL output is suppressed by approximately 20 dB by passing the laser light through a solid silica, Fabry-Perot etalon (FSR = 15.14 GHz, finesse = ~32, LightMachinery). A small amount of laser light (~4%) is picked off following the etalon and measured with a photodetector so that the ECDL center frequency can be actively locked to a transmission peak of the etalon. The resulting fluctuations in laser intensity after locking are < 5%. Locking the laser to the etalon also reduces wavelength drift of the ECDL output, such that the absolute stability is ± 20 MHz over 5 minutes, limited by temperature-induced/mechanical drift of the etalon itself [7]. An optical isolator (IO-5-780-VLP, Thorlabs) is placed directly following the laser output port to prevent back-reflections from entering the laser.

Human interface

Laser light is carried to the human interface via polarization-maintaining single-mode optical fiber (PM780-HP, Thorlabs), where it is collimated and then split into sample and reference arms using a half-wave plate and polarizing beam splitter (CM1-PBS252, Thorlabs). In the sample arm, laser light is focused onto the cornea using a microscope objective (20x, effective NA = 0.1, Mitutoyo). In the reference arm, laser light is focused onto two known reference materials: polystyrene and water at room temperature, through an achromatic doublet lens (f = 30mm, Thorlabs). The temperature of the reference materials is monitored and saved in real-time so that temperature calibration can be applied to the reference spectrum. In both sample and reference arms, a quarter-wave plate is inserted so that back-scattered light is transmitted through the output port of the polarizing beam splitter, where it can then be coupled into a single-mode optical fiber (780HP, Thorlabs) for transmission to the spectrometer. The human interface is mounted to a stripped-down slit lamp headrest with joystick-based spatial manipulator allowing for manual adjustment of the lateral scanning location on the cornea. The objective lens is mounted on a motorized translation stage (T-LSM025A, Zaber Technologies), allowing for automated axial scanning through the corneal depth. At normal incidence, specular reflection significantly diminishes the signal-to-noise ratio of the Brillouin spectrum. Thus, it is necessary to tilt the human interface by about 15° with respect to the head rest, and additionally have the subject fixate their gaze on a target making an angle of about 10° (in the opposite direction) with respect to the head rest, so that the incidence angle is > 10° over a central zone of ~∅6 mm laterally across the cornea.

For eye tracking, a light emitting diode (940 nm, ~2 mW on sample surface, M940L3, Thorlabs) is used. The near-infrared range is chosen because it reduces the sensitivity of intensity-based pupil-detection algorithm to eye color. The eye is imaged using a high-resolution, monochrome CMOS camera (Mako G-419B, Allied Vision). A long-pass filter is used to reject the laser line and background room lights. Frames are captured on the CMOS camera synchronously with the EMCCD (but at a higher frame rate) so that events such as blinking or eye movement can be identified. Because the relative positions of the objective lens and CMOS camera are fixed, the laser location on the CMOS camera is stationary. Hence, the lateral coordinates for each axial Brillouin scan be determined relative to the pupil center by detecting the pupil location in each CMOS camera image.

Spectrometer

The spectrometer design is based on two virtually imaged phase array (VIPA) etalons in a cross-axis configuration, similar to that reported previously [8,9]. The two VIPAs used are identical (R1 = 99.9%, R2 = 95%, tilt angle ≈ 1.5°, LightMachinery). A series of lenses (cylindrical lenses C1 and C2, plus spherical lenses A1 and A2) with focal length 200 mm are used to focus light onto the VIPAs, where angular

dispersion occurs, and to relay light to two spatial filters (S1 and S2), which block undesired spectral components (Rayleigh peaks). The gradient intensity filter (G) following the first VIPA (V1) enhances the spectral contrast by reshaping the intensity profile along the dispersion direction [10]. The first VIPA (V1) is oriented vertically, while the second VIPA (V2) is oriented horizontally, resulting in a net frequency dispersion along the diagonal axis, which is imaged onto a diagonally-oriented, electron-multiplying charge-coupled detector (EMCCD, iXon 897, Andor Technologies) using a telescope (T). In the portable system, additional folding mirrors are used to make the spectrometer more compact. Typical Brillouin signal counts detected at the EMCCD are on the order of 900 photons/s, requiring exposure times of 100 – 400 ms. The rate of spectrum dispersion on the EMCCD is ~0.125 GHz/pixel.

Table S1. Specifications of the Brillouin Systems

**Parameter**	**Value**
Laser wavelength	780 ± 0.2 nm
Laser power on the cornea	3 – 5 mW
Data acquisition time per point	0.2 – 0.4 s
Transverse resolution	~ 5 µm
Axial resolution	~ 35 µm
Spectral sensitivity	~ ± 8 MHz
Measurement repeatability	~ ± 10 MHz

Spectrometer frequency calibration

To calibrate the spectrometer, we used two materials with known Brillouin shifts, water and Polystyrene (PS). Frequency shifts of the two materials at room temperature were pre-determined with calibrated spectrum dispersion, as Ω_{Water} = 5.11 GHz and Ω_{PS} = 9.62 GHz, respectively. Spectral dispersion rate is then determined by:

$$\mathrm{SD} = 2 * \frac{\Omega_{\mathrm{PS}} - \Omega_{\mathrm{water}}}{(\mathrm{P_{PS-B}} - \mathrm{P_{PS-A}}) + (\mathrm{P_{W-B}} - \mathrm{P_{W-A}})}$$

where $\mathrm{P_{PS-A}}$ and $\mathrm{P_{PS-B}}$, $\mathrm{P_{W-A}}$ and $\mathrm{P_{W-B}}$ are paired peaks in the reference signal from PS and water, respectively (Fig. S3d). Precise free spectral range during a measurement is estimated as:

$$\mathrm{FSR} = 2 * \Omega_{\mathrm{PS}} - |\mathrm{P_{PS-B}} - \mathrm{P_{PS-A}}| * \mathrm{SD}$$

Sample frequency shift Ω_x is defined as the distance along the spectrum line from the Rayleigh peak (laser line, blocked by optical shutter and therefore not visible in this figure) to the fitted signal peak, and is given by (Fig. S3b):

$$\Omega_x = 0.5 * [\mathrm{FSR} - (P_2 - P_1)\,]$$

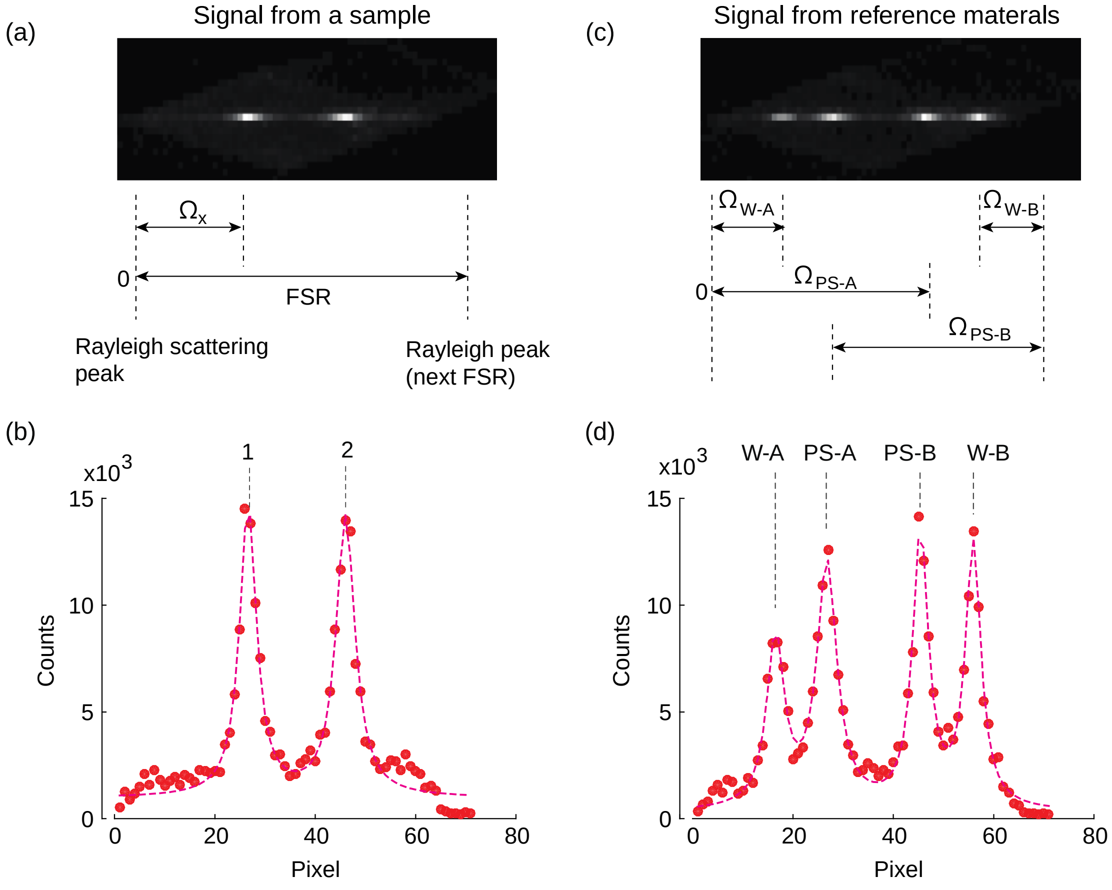

Fig. S3: Spectrometer frequency calibration. (a) EMCCD recording of a typical sample signal. (b) Plot of signal intensity across spectrum line from (a) and resulting fit to a Lorentzian function. Dots: pixel intensity values; dashed line: fitting results. (c) EMCCD recording of a typical signal from reference materials, water and Polystyrene (PS) by focusing light on the water-PS interface. (d) Plot of signal intensity across spectrum line from (c) and the resulting fit to a Lorentzian function. Dots: pixel intensity values; dashed line: fitting results.

Signal-to-noise ratio (SNR) and measurement sensitivity

For a CCD detector, shot noise is ideally the primary contributor to signal-to-noise ratio (SNR), with negligible contribution from dark current and readout noise (operation in the so-called, shot-noise-limited regime). However, in detectors using amplifying technologies, such as EMCCDs, the signal multiplying process increases the degree of variance in the signal around the mean value. Technically the detectors work in the shot-multiplicative-noise-limited regime in the best case. For simplicity, we still use the term 'shot-noise-limited regime'. Our spectrometer reading is background free and works in this regime. To verify this, we measured the SNR at different illumination energy levels on a piece of poly(methyl methacrylate) (PMMA) material, using 5 mW input power and integration time increasing from 50 to 600 ms. For each illumination level, 50 measurements were obtained continuously at a single location on the sample. SNR is defined as the ratio of mean signal intensity to standard deviation of signal intensity. A good square root dependence of SNR on the product of illumination power and integration time (total number of photons collected) was found, suggesting shot-noise limited operation (Fig. S4a). In this regime, the measurement accuracy is ultimately limited by the SNR. To find an optimal operating point, we plot measurement errors (standard deviation of measurements) versus 1/SNR. An EMCCD integration time of 0.2 s corresponds to standard deviation of <10 MHz at 5 mW. In Fig. S4c, we show the resulting distribution of estimated Brillouin frequency shifts using these settings (5 mW, 0.2 s) when the measurement is repeated 400 times continuously. The standard deviation of these measurements is ± 7.8 MHz. This corresponds to a relative measurement uncertainty of ~ 0.1 to 0.2%.

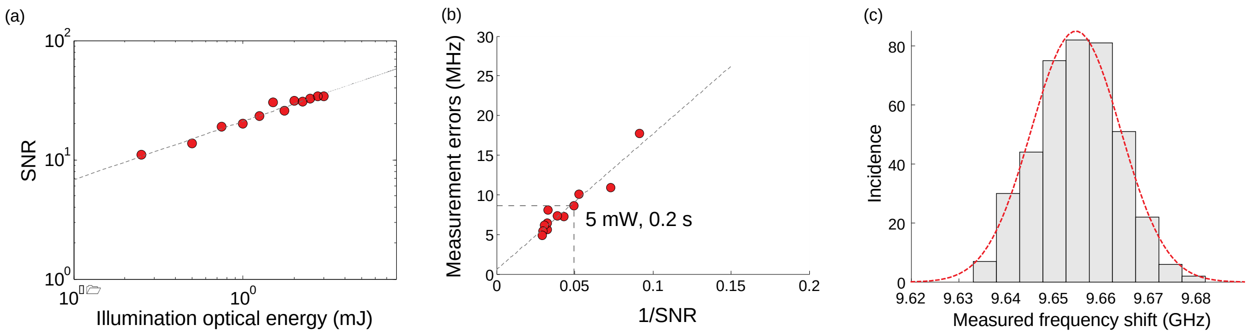

Fig. S4: Signal to noise ratio and measurement sensitivity. (a) Plot of signal-to-noise ratio versus optical energy. Line: linear fit. (b) Plot of measurement error (based on standard deviation of 5 consecutive measurements) versus inverse SNR. Line: linear fit (c) Histogram of estimated Brillouin values over 400 consecutive measurements. Standard deviation is ± 7.8 MHz.

Spatial resolution

Light delivery to and light collection from the sample is arranged in a confocal configuration. The foci for light delivery and light collection overlap, hence the spatial resolution is determined by the focal power of the objective lens. Lateral resolution is calculated to be approximately $R_{lateral} \approx 5$ μm. We experimentally determine axial resolution. An edge spread function $\mathrm{ESF(z)}$ was obtained via axial scanning though the plastic-water interface of a PMMA cuvette, with a step size of 20 μm. The axial resolution is estimated to be $\mathrm{R_{axial}} = \frac{\mathrm{d}}{\mathrm{dx}}\mathrm{ESF(z)} \approx 35$ μm, given by the FWHM of the fitted Gaussian profile to the ESF (Fig. S5b).

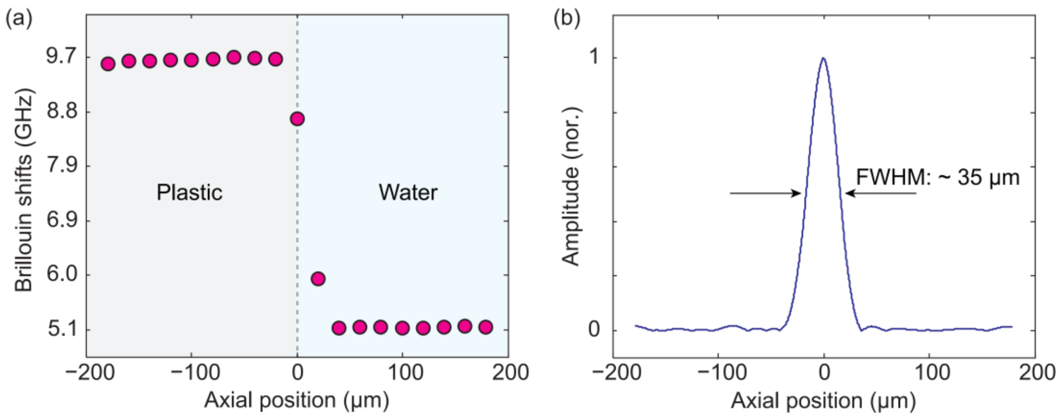

Fig. S5. Axial resolution. (a) Axial scanning profile of a PMMA-water interface. (b) Fitted Gaussian function to the 1st order derivative of the interface edge spread function ESF, from which we estimate the axial resolution of the system to be around 35 μm.

Temperature dependence of Brillouin measurements and calibration

Brillouin frequency shift is temperature-dependent, so temperature fluctuation of the reference materials will cause error in our calibration, and hence our estimation of the Brillouin frequency shift of the sample. We studied the temperature dependence of the reference materials and applied a temperature correction in post-processing to alleviate its effect on measurements. We first measured Brillouin frequency shift in water versus temperature and compared with theoretical prediction (Fig. S6a), calculated using $\Omega = 2n\frac{v_{water}}{\lambda}\sin(\beta)$, where n is the refractive index of water, v_{water} is the speed of sound in water, λ is laser wavelength, which in this study was 780 nm, and $\beta = 180°$ is the scattering angle. Refractive index and speed of sound in water at different temperatures are taken from literature [11]. Speed of sound is calculated following experimental reports by Bashkatov and Genina [12], which is valid from 0 – 100°C. Distilled water was pre-heated to > 80°C, and data was acquired as the water cooled down to room temperature. 10 consecutive Brillouin measurements were taken with 5 mW input power and ~ 0.7 s integration time at each temperature, which was measured with a high precision thermometer (HH804U, Omega). Temperature drift occurred during in vivo data acquisition. To correct for this, we quantified the temperature-induced measurement variations within a narrower temperature window ranging from 20 – 30 °C (corresponding to the maximum temperature range in a clinical setting), and created a lookup table containing Brillouin frequency shift of the reference materials at different temperatures by fitting 2nd polynomials to the data (Fig. S6b). The table was then used to correct the calibration data taken for each axial scan.

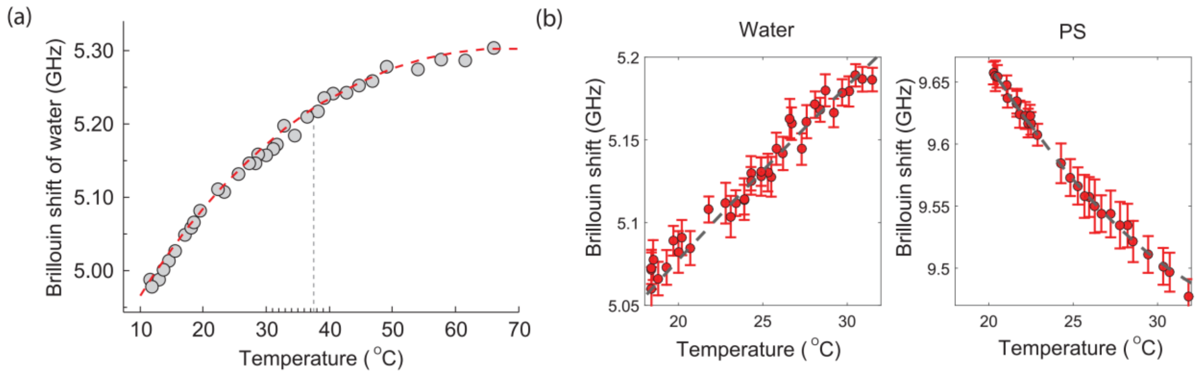

Fig. S6: Temperature dependence of Brillouin measurements and calibration. (a). Brillouin frequency shift of water versus temperature with theoretical prediction. Dots: raw data; red dashed line: prediction. (b) Temperature dependence of Brillouin frequency shift in water and PS over the 20 – 30°C window for temperature correction.

Brillouin measurement short-term and long-term stability

Both short-term and long-term measurement instability may be caused by environment-induced light source and/or spectrometer drift, mechanical instability of the optical system, etc. To minimize these effects, the imaging system was calibrated daily to maintain its performance. Experiments were also carried out to quantify the measurement variations of the system. In laboratory conditions with typical temperature drift up to 1°C per hour, we characterized short term measurement stability through 12 consecutive measurements of the Brillouin frequency shift of a water sample, taken at intervals of 2.5 minutes. At each of the 12 time-points, the Brillouin frequency value is itself obtained from the average of 5 measurements. Averaged values lie within a ±5 MHz range above and below baseline (Fig. S7a). To examine day-to-day variation, the Brillouin frequency shift of a PMMA sample was measured over four consecutive days at the same time of day in the laboratory environment. 5 measurements were averaged for each daily Brillouin value. Measurements were obtained with 0.2 s integration time at 5 mW input optical power. Day-to-day variation is found to be < 10 MHz (Fig. S7b). On a longer timescale, Brillouin measurements of water over a 140-day period indicate that the long-term stability is ~ ±10 MHz (Fig. S7c).

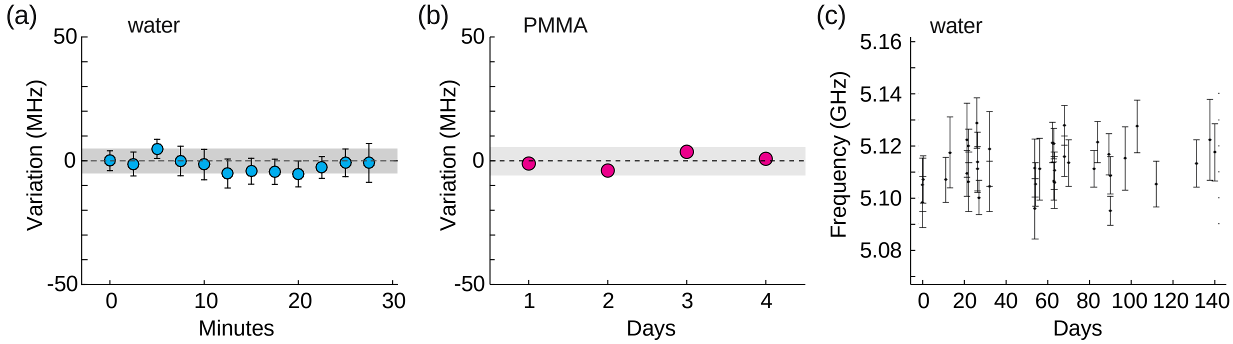

Fig. S7. Measurement of short-term and long-term stability. (a) 12 measurements of water, taken 2.5 minutes apart in the laboratory environment. Dots: mean of 5 measurements; Error bar: standard deviation of 5 measurements; Shadowed zone: ±5 MHz window around the baseline. (b) Brillouin measurements of a PMMA sample over four consecutive days, taken at the same time of day in the laboratory environment. Dots: mean of 5 measurements; Error bar: standard deviation of 5 measurements; Shadowed zone: ±5 MHz window around the base line. (c) Repeated Brillouin measurements of water over a 140-day period, yielding ±10 MHz long-term stability.

Daily variations in in vivo Brillouin measurements

We performed preliminary experiments to assess the daily variation of measurements for individual subjects. For this, we measured Brillouin frequency shift of 3 healthy subjects' corneas over 3 consecutive days at the same time of day (around 5 o'clock pm) in a laboratory with no environmental (temperature, humidity) control. Before Brillouin measurements, central corneal thickness was measured with corneal topography. Standard deviation of CCT measurements of subjects 1, 2, and 3 were: 4.7, 1.0, and 5.5 µm, respectively (Fig. S8a). 5 measurements taken in the central cornea with a radius (R) of < 1 mm from pupil center were averaged to obtain each daily Brillouin frequency-shift value. Standard deviation of the measurements from subjects 1, 2, and 3 were 0.0042, 0.0062, and 0.0169 GHz, respectively (Fig. S8b). Mean standard deviation of Brillouin frequency shift from the three subjects was 0.009 GHz, which is within the sensitivity of our instrument.

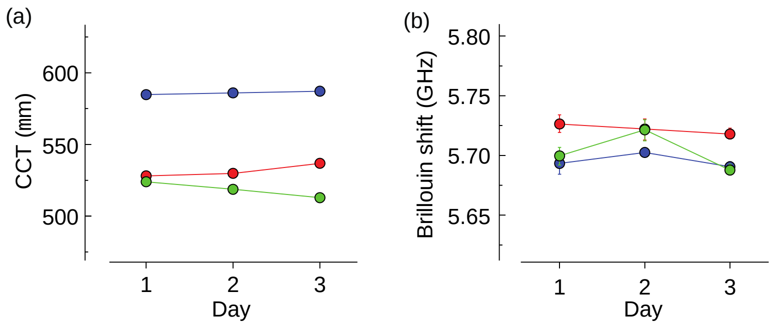

Fig. S8. Daily variation of *in vivo* measurements. (a) Central corneal thickness of three healthy subjects over three consecutive days at 17:00, measured using corneal Scheimpflug topography. (b) Brillouin frequency shift measurements from the same subjects color-coded as in (a). Dots: average of 5 central measurements; error bars: standard deviation of the 5 central measurements.

Definition of zones for analyzing spatial variation in Brillouin measurements

To analyze the spatial variation in Brillouin values across the cornea, we define various regions of interest (see Fig. S9 for a graphical illustration of these zones). Central region is defined as the zone within a R = 1 mm radius around the pupil center; Peripheral region in contrast is the area with a distance R > 3 mm from the pupil center. In keratoconic corneas, the 'cone region' is defined as the area with R < 1 mm from the thinnest point (the 'cone center') identified with Scheimpflug-based corneal topographic imaging, and the 'outside-cone region' refers to the region R > 3 mm from the cone center.

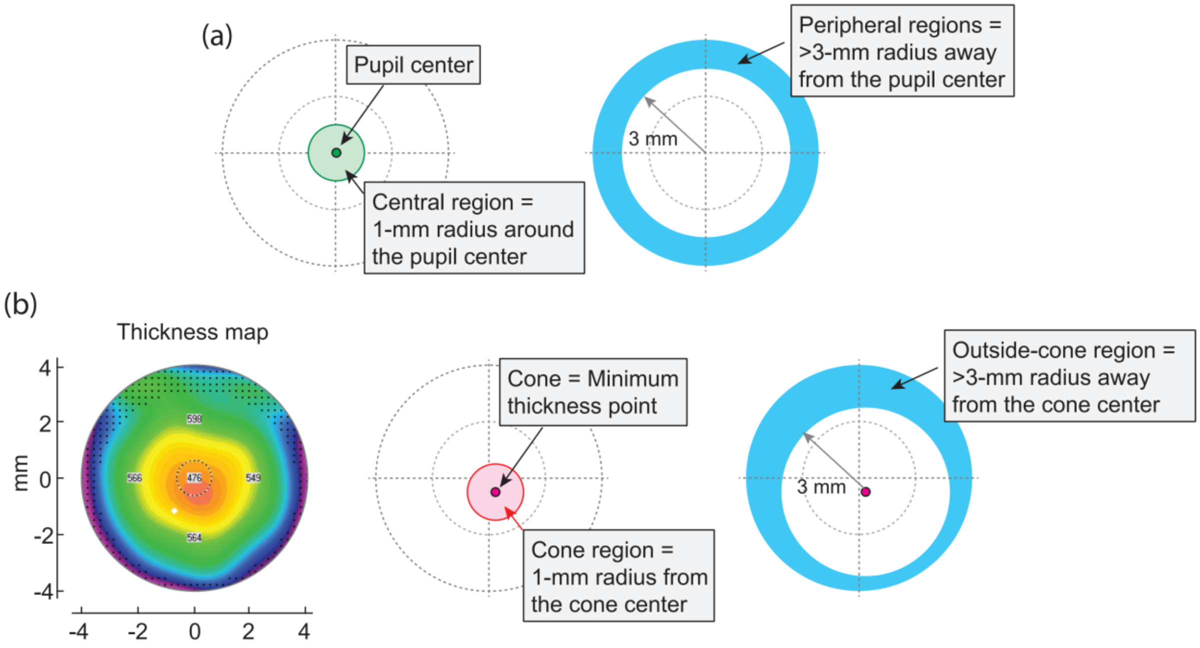

Fig. S9. Definition of zones for analyzing spatial variation. (a) Central region and the peripheral regions, which are defined as areas on the corneal surface within R < 1 mm and R > 3 mm, respectively. (b) The 'cone region' and 'outside-cone region', defined as areas within R < 1 mm R > 3 mm from the 'cone center' that is the thinnest point identified by Scheimpflug corneal topography, respectively.

Regional differences in Brillouin frequency shift measured in keratoconic corneas

We defined annular regions extending radially outwards in 0.5 mm increments, centered on the cone (again specified by the thinnest point in the corneal topography). The Brillouin frequency shift in the corneal stroma was averaged over the spatial extent of these annuli and then the difference was taken with respect to the value in the cone region (<1 mm from the thinnest point). Results from the normal cornea group, and KC stage I – IV groups are presented in Fig. S10.

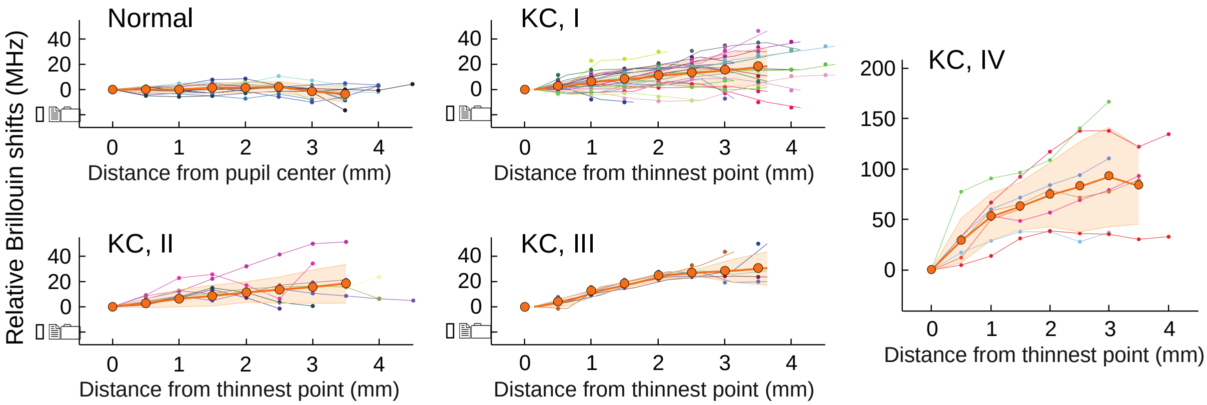

Fig. S10. Regional differences in Brillouin frequency shift measured in corneas. Plots show lateral profiles of average Brillouin frequency shift from cone center to peripheral regions in normal, and KC stage I – IV corneas.

Receiver operating characteristic curves

To estimate the predictive value of the Brillouin indices, we computed receiver operating characteristic (ROC) curves for four metrics: Brillouin shifts in the cone, Brillouin regional difference between the cone and out-of-the-cone regions, and two corneal topography parameters, minimum corneal thickness, 'min T', and maximum sagittal curvature, 'K_{max}'. The data were fitted to a binary classification model based on the "gold-standard" diagnosis made by the physician experts in IROC. The prediction outcome was labeled as positive (P, with KC) or negative (N, without KC). The ROC curves were generated by calculating the true positive rate (TPR, or sensitivity), as $\mathrm{TPR} = \mathrm{TP}/(\mathrm{TP} + \mathrm{FN})$, where TP and FN are true positive rate and false negative rate. False positive rate, 1-specifity, for different criterion values was calculated with $\mathrm{FPR} = 1 - specifity = \mathrm{FP}/(\mathrm{TN} + \mathrm{FP})$, where FP and TN are false positive rate and true negative rate, respectively. Figures S11 shows the ROC curves for mild KC cases (Stage-I, n=25). The area under the curve (AUC) reflects the capability of a particular parameter in discriminating between the normal and disease cases. AUC is 0.92 for mean Brillouin value of the cone and 0.85 for Brillouin regional difference, which are higher or similar to 0.77 for minimum thickness and 0.87 for K_{max}.

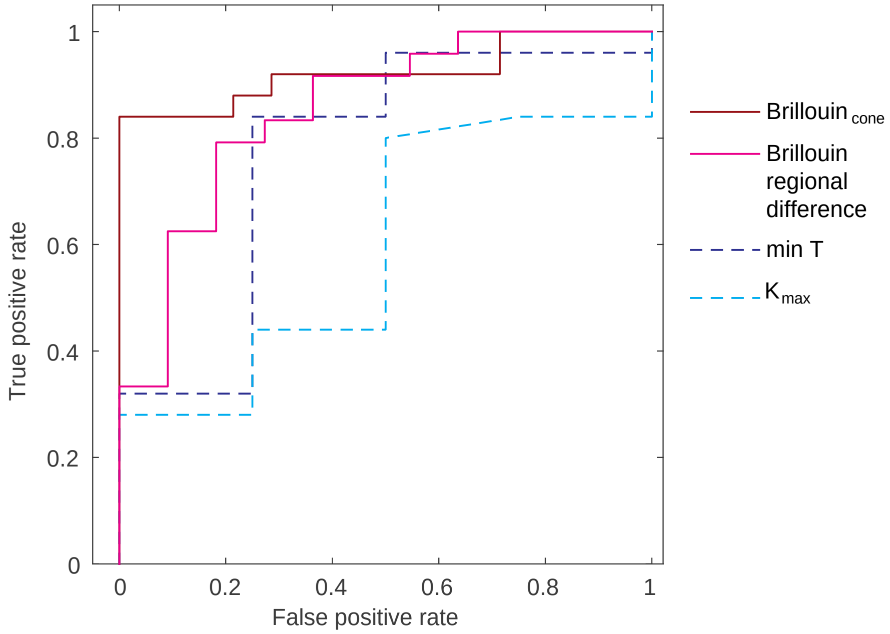

Fig. S11. The receiver operating characteristic (ROC) curves for mean Brillouin shift in the cone (dark red solid line) and Brillouin shift difference between the cone and outside-the-cone regions (magenta solid line) in comparison to maximum sagittal curvature K_{max} (cyan dashed line) and minimum corneal thickness (dark blue dashed line).

Safety of Brillouin measurements

Our Brillouin system uses illumination intensity levels comparable to or less than equivalent ocular instruments that are already FDA-cleared and widely used in clinical practice, such as corneal confocal scanning microscopy and anterior-segment optical coherence tomography. The optical source in our Brillouin instrument emits near-infrared light at a wavelength of 780 nm. Exposure of human eyes to this light during our study was within safety limits established by the American National Standard for Safe Use of Lasers (ANSI Z136.1-2007). The ANSI standard indicates that exposure to 780 nm laser light at the level we use for imaging (5 mW) does not pose a risk to the subject if the continuous exposure does not exceed 5.75 minutes. In our study, the continuous exposure time for each axial scan was < 15 s.

References:

[1] L. Brillouin, Ann. Phys. (Paris). **9**, 88 (1922).

[2] J. G. Dil, Reports Prog. Phys. **45**, 285 (1982).

[3] S. Dennis, S. Khan, and K. M. Meek, Biophys. J. **85**, 2205 (2003).

[4] S. Patel, J. L. Alió, and J. J. Pérez-Santonja, Investig. Ophthalmol. Vis. Sci. **45**, 3523 (2004).

[5] B. Fabry, G. N. Maksym, J. P. Butler, M. Glogauer, D. Navajas, and J. J. Fredberg, Phys. Rev. Lett. **87**, 1 (2001).

[6] I. L. Fabelinskii, *Molecular Scattering of Light* (1968).

[7] P. Shao, S. Besner, J. Zhang, G. Scarcelli, and S.-H. Yun, Opt. Express **24**, 22232 (2016).

[8] G. Scarcelli and S. H. Yun, Opt. Express **19**, 10913 (2011).

[9] G. Scarcelli, P. Kim, and S. H. Yun, Opt. Lett. **33**, 2979 (2008).

[10] G. Scarcelli, W. J. Polacheck, H. T. Nia, K. Patel, A. J. Grodzinsky, R. D. Kamm, and S. H. Yun, Nat. Methods **12**, 1132 (2015).

[11] N. Bilaniuk and G. S. K. Wong, J. Acoust. Soc. Am. **93**, 1609 (1993).

[12] A. N. Bashkatov and E. A. Genina, Proc. SPIE **5068**, 393 (2003).
